# Research on digital tool in cognitive assessment: a bibliometric analysis

**DOI:** 10.3389/fpsyt.2023.1227261

**Published:** 2023-08-23

**Authors:** Leian Chen, Weizhe Zhen, Dantao Peng

**Affiliations:** ^1^China-Japan Friendship Hospital (Institute of Clinical Medical Sciences), Chinese Academy of Medical Sciences & Peking Union Medical College, Beijing, China; ^2^Department of Neurology, China-Japan Friendship Hospital, Beijing, China; ^3^Graduate School, Beijing University of Chinese Medicine, Beijing, China

**Keywords:** neuropsychological tests, cognition, computerized assessment, digital cognitive assessment, bibliometric analysis

## Abstract

**Objective:**

The number of research into new cognitive assessment tools has increased rapidly in recent years, sparking great interest among professionals. However, there is still little literature revealing the current status and future trends of digital technology use in cognitive assessment. The aim of this study was to summarize the development of digital cognitive assessment tools through the bibliometric method.

**Methods:**

We carried out a comprehensive search in the Web of Science Core Collection to identify relevant papers published in English between January 1, 2003, and April 3, 2023. We used the subjects such as “digital,” “computer,” and “cognitive,” and finally 13,244 related publications were collected. Then we conducted the bibliometric analysis by Bibliometrix” R-package, VOSviewer and CiteSpace software, revealing the prominent countries, authors, institutions, and journals.

**Results:**

11,045 articles and 2,199 reviews were included in our analyzes. The number of annual publications in this field was rising rapidly. The results showed that the most productive countries, authors and institutions were primarily located in economically developed regions, especially the North American, European, and Australian countries. Research cooperation tended to occur in these areas as well. The application of digital technology in cognitive assessment appealed to growing attention during the outbreak of the COVID-19 epidemic.

**Conclusion:**

Digital technology uses have had a great impact on cognitive assessment and health care. There have been substantial papers published in these areas in recent years. The findings of the study indicate the great potential of digital technology in cognitive assessment.

## 1. Introduction

Around 55 million people worldwide live with dementia, and this number is set to rise to 139 million by 2050 (1). Besides the ever-increasing number of patients, it is worth attention that up to three-quarters of those with dementia worldwide have not received a diagnosis (1). The clinical diagnosis of dementia or mild cognitive impairment is based on a comprehensive assessment framework backed with neurocognitive, neuroradiological, and biochemical evidence (2, 3). As the core clinical criteria for dementia, cognitive screening and monitoring are helpful to formulate clinical treatment plans and determine clinical staging, thus providing an objective basis for clinical diagnosis and treatment (4). Standardized cognitive tests are recommended by some official guidelines to diagnose cognitive disorders and assess their severity (5). A formal neuropsychological assessment always consists of the assessment for various domains of cognitive functions, including memory, language, attention, or executive function.

Traditional cognitive measures, the most common approach in clinical practice, are based on paper-and-pencil assessments. Nowadays, modern health care and medicine are characterized by continuous digital innovation (6). Technological advances with transformative potential prompt the evolvement of cognitive assessment instruments. Digital cognitive assessment technology facilitates repeated and continuous assessments and the collection of clinical data, much more convenient and cost-effective than paper-and-pencil assessments (7). Moreover, the growth of older adult tech-adoption and the outbreak of the COVID-19 pandemic necessitate digital cognitive assessment (8). Digital technology uses in cognitive management are thriving and gaining in popularity. This year, China issued clinical practice guidelines targeting the application of electronic assessment tools and digital auxiliary equipment to the management of cognitive disorders (9). This guideline paves the way for health care about cognition from assisting with diagnosis, recording results, clinical follow-up, and patient education, to referral and consultation. Given the numerous related publications and the growing research interest, it is essential to critically examine and analyze the existing studies to gain a comprehensive understanding of these domains.

Bibliometric analysis is an objective and quantitative method for exploring and analyzing large volumes of scientific data in rigorous ways (10). It evaluates research impact, identifies gaps that require further research, and provides a useful tool for decision-making in academia and industry. In the field of medical healthcare, bibliometric analysis enables researchers, clinicians, and healthcare policymakers to collect information about a specific field and shed light on the emerging areas in that field, while promoting interdisciplinary collaborations (10–13).

Many studies have used bibliometric methods to study progress in the application of digital technologies in medicine or health care (6, 14). There is, however, a paucity of studies providing a holistic snapshot of advances in digital technology on cognitive management, not to mention the cognitive assessment tools, regardless of numerous published literature on this scientific area. The aim of this study was to provide a comprehensive picture of the research related to digital cognitive tests from 2003 to 2023 by bibliometric methods.

## 2. Mini literature review

### 2.1. Background

The American Academy of Clinical Neuropsychology (AACN) and the National Academy of Neuropsychology (NAN) define digital neuropsychological assessment tools as devices which utilize a computer, digital tablet, handheld device, or other digital interface instead of a human examiner to administer, score, or interpret tests of brain function and related factors relevant to questions of neurologic health and illness (15). Patients perform the cognitive tests in the man–machine interface through a keyboard, voice, mouse, or touch screen, instead of directly interacting with a person. The use of computerized devices increases the accessibility to neuropsychological assessment for patients when professional neuropsychological services are scarce. These potential advantages accelerate the use of computerized testing in research, clinical trials and clinical practice. In addition to technology, society influences the practice of cognitive assessment as well. Although the AACN and the NAN outlined appropriate standards for the development of digital assessment tools development back in 2012 (15), the field had not significantly advanced as expected for the next years (16). The COVID-19 pandemic occasionally imposed the halt of scheduled clinical activities, forcing clinicians to arouse a renewed interest in the use of digital tools as alternative strategies (17). On the other hand, due to the various digital technologies for management rolled out by the government during the pandemic, many older adults had access to digital services. In the first 3 months after the outbreak of the COVID-19, the number of middle-aged and older adult internet users in China increased by 61 million, a number that had not been achieved in the prior 10 years (18). Familiar with digital devices, people are prone to accept the computerized cognitive assessments.

### 2.2. The category of digital cognitive assessment

**Figure 1:**
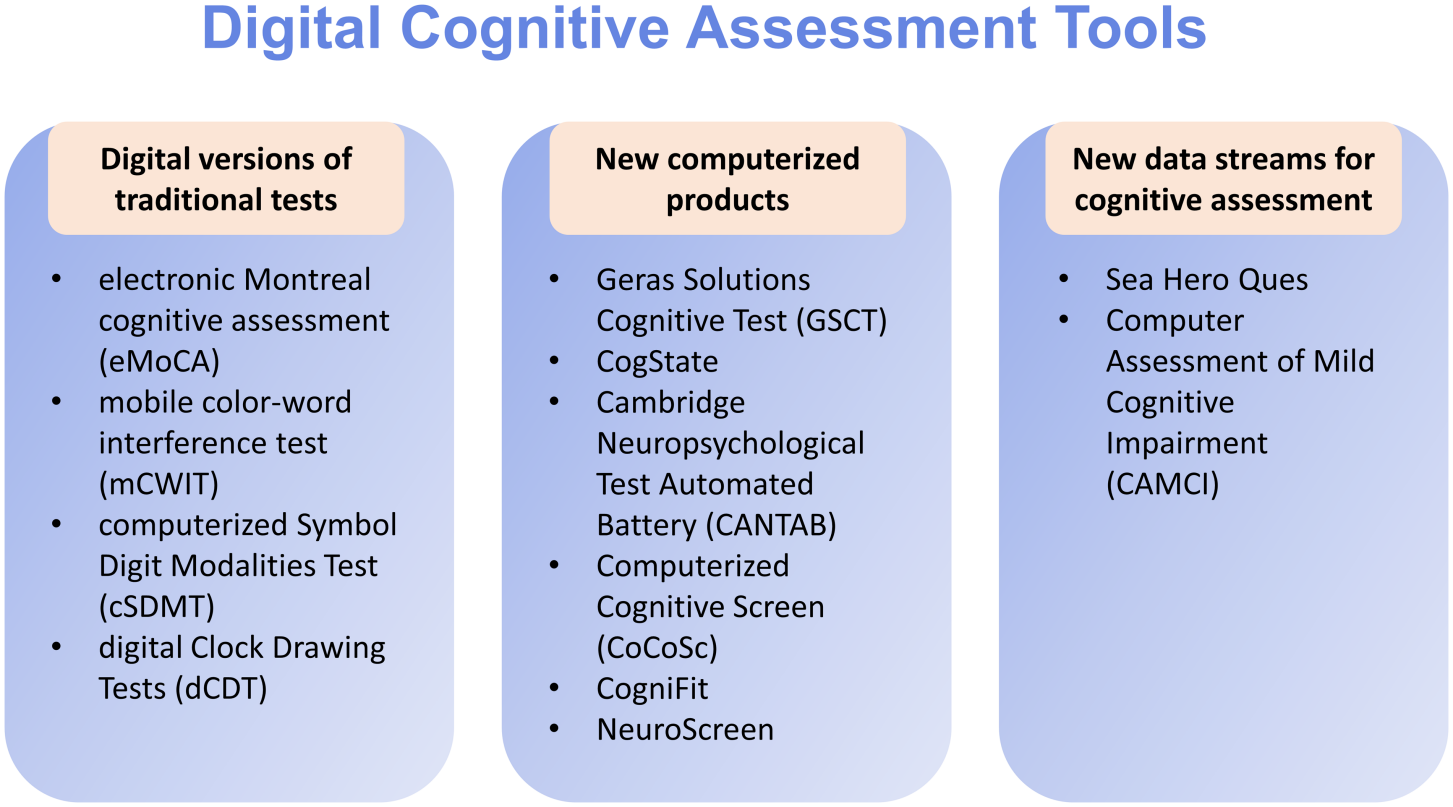
Some digital cognitive assessment tools.

The main cognitive assessment tools on the market are divided into three categories (7) (Figure 1). The first category is the digital version of the existing pen-and-paper conventional tests, like the electronic version of the Montreal cognitive assessment (eMoCA) (19) and the digital Clock Drawing Tests (dCDT) (20, 21). These traditional cognitive measures are programmed for computer administration, and often target specific cognitive domains (15).

Second is new computerized neuropsychological products or new test batteries (22, 23), which are specifically designed for screening, comprehensive assessment or diagnostic purposes (16). These assessment tools, targeting several cognitive domains, include the Geras Solutions Cognitive Test (GSCT) (22), CogState (24), Computerized Cognitive Screen (CoCoSc) (25), Inoue (26) and the Cambridge Neuropsychological Test Automated Battery (CANTAB) (27).

The last is the use of new data streams for cognitive assessment specially designed for computers or other mobile platforms, always embedded with new types of technologies (7). Some games are developed based on the technology of virtual reality (VR) and spatial navigation (8). By watching players’ performance on tasks of various complexity in virtual space during these games, the researchers could measure participants’ cognitive function (28). For example, Sea Hero Quest, a video game designed for aiding the early diagnosis of Alzheimer’s disease, quantifies impairments in navigation performance. Participants were required to navigate a boat to the goal locations in a virtual environment like lakes or rivers. Navigation ability in the real world can be predicted from the results according to performance in this game. More importantly, the validity of this game has been proved in the two cities, London and Paris (29).

### 2.3. Current challenge

Digital cognitive assessment tools have been developed to replace and improve upon traditional cognitive measures, but their development remains in the early stages (30). A range of issues need to be solved. First, high concordance may not be guaranteed after simply converting traditional pen-and-paper tests into the digital versions (15, 16). Once a pen-and-paper test has been digitized, it has become a new one. Researchers must investigate the equivalence of computer tests and paper-and-pencil tests (31). Second, for new tests or batteries, the majority of present studies still focus on their reliability and validity, with small and limited samples (7). Third, rapid obsolescence of particular tests and test norms has to be considered as a result of fast iterations over time in both hardware and software (32). Additionally, various and disparate psychometric analyzes make comparison extra difficult, as well as data transmission, especially across digital device classes. Clinicians always have difficulty interpreting the reported results from different new neuropsychological tests. Therefore, diagnostic errors and poor clinical decisions could potentially arise. Another important consideration is that their application scenarios are narrowly focused on a few specific places, like hospitals, community clinics, psychological counseling institutions, physical examination centers and specialized experience centers (18). The limited application scenarios seriously restrict user accessibility and reduce adherence. These difficulties hinder the widespread application of digital devices for the detection, diagnosis and monitoring of cognitive disorders (16).

## 3. Methods

### 3.1. Search strategy

The Web of Science Core Collection (WoSCC) was selected as the data source in this study. The WoSCC database is a comprehensive, systematic, and authoritative database with approximately 10,000 prestigious and high-impact academic journals (12). The WoSCC database has now been commonly used for bibliometric analyzes and scientometric visualization (33). The search for potential publications was conducted on April 5, 2023. The search term “cognitive assessment” was searched in the Medical Subject Headings (MeSH: https://www.ncbi.nlm.nih.gov/mesh) of PubMed to obtain related MeSH terms. Other vocabularies in our search were derived from the published literature or based on common knowledge. Then these terms were used to perform advanced searches on WoSCC, with editions set as SCI-EXPANDED and SSCI. The publication time ranged from January 1, 2003 to April 3, 2023. The type of documents was confined to “articles” and “reviews” published in English. The retrieval results were exported in plain text format and tab-delimited file with the content “Fully Recorded and Cited References.” The detailed search strategy is presented in Supplementary Appendix.

### 3.2. Data analysis

**Figure 2:**
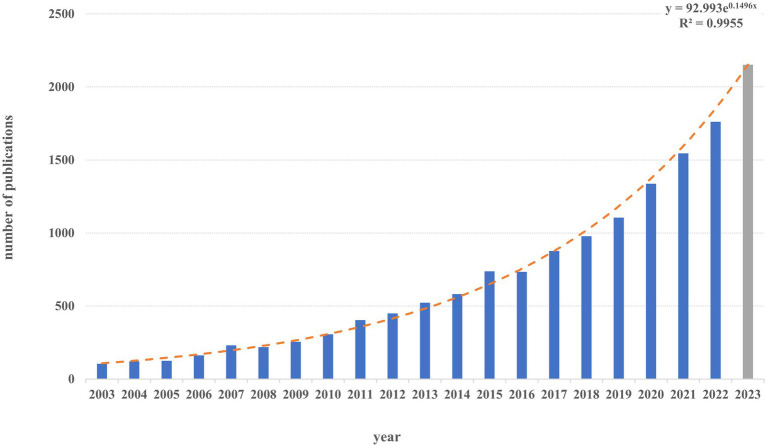
Distribution of the annually published documents from 2003 to 2022. y is the annual publications and x is the year rank.

Bibliometric analysis was conducted in this study with the aim to provide a holistic view of digital neurocognitive tests. The following characteristics were described: annual publications and their trends, as well as the most prolific countries/regions, institutions, authors and their cooperation networks. To analyze research status and current hotspots, the study also described clustered networks of co-cited references and keywords, and identified the keywords and references with the highest citation bursts. Data were mostly visualized by three tools, namely, “Bibliometrix” R-package (R version 4.1.2; Bibliometrix version 4.1.2) (34, 35), VOSviewer (version 1.6.19) (36), and CiteSpace (version 6.1. R6) (37). All information on the data has been exported into Microsoft Office Excel or R software to analyze. The annual publications and growth trends (Figure 2) were generated by Microsoft Excel 2019 (38). “Bibliometrix,” a package developed in the R language, was used to conduct basic bibliometric analyzes, such as summarizing the number of publications, the most productive countries, sources, affiliations and authors. VOSviewer is a software tool for visualizing and exploring network data in bibliometric analysis (36). In this study, data were imported into VOSviewer for further detailed analyzes like creating a co-authorship network and clustering the authors’ keywords. VOSviewer was also used to identify the top 20 recurring author keywords and references. CiteSpace, produced by Chaomei Chen (37), was used to perform reference cocitation and burst analyzes. In this study, NP stands for the numbers of publications, and TC stands for total citations. Cluster analysis results were evaluated using the Modularity (Q-score) and Silhouette (S-score) coefficients. The Q-score measures network clusters, and a score greater than 0.3 indicates significant clustering. The S-score confirms consistency among data clusters, with coefficients of 0.3, 0.5, or 0.7 indicating a homogeneous, reasonable, or extremely credible network, respectively (39).

## 4. Results

### 4.1. The basic condition of digital cognitive assessment

#### 4.1.1. Publication summary

A total of 13,244 records about digital assessment tools of cognition were published from January 2003 to April 2023, including 11,045 articles and 2,199 reviews. We counted the number of annual papers in this field. Figure 2 shows the number and trend of the annual publications. The annual growth rate was 5.51%. As is shown in Figure 2, during the past two decades, the number of articles has increased exponentially, growing from 104 articles in 2003 to 1,761 in 2022. Between 2003 and 2012, the annual paper output for electronic cognitive testing was less than 500, whereas the number of annual publications in 2019 exceeded 1,000 for the first time, showing the rapid growth in the research of electronic measures for testing cognition. Additionally, the fitting curve reflects an exponential relationship between the number of articles and the publication year (excluding 2023) (R^2^ = 0.996). If this exponential growth continued, there would be roughly 2,152 related papers published in 2023. It appears that digital cognitive assessment is one of the popular hot topics that attract a lot of interest from academics. Other information can be found in Supplementary Appendix.

#### 4.1.2. Analysis of countries/regions and institutions

**Figure 3:**
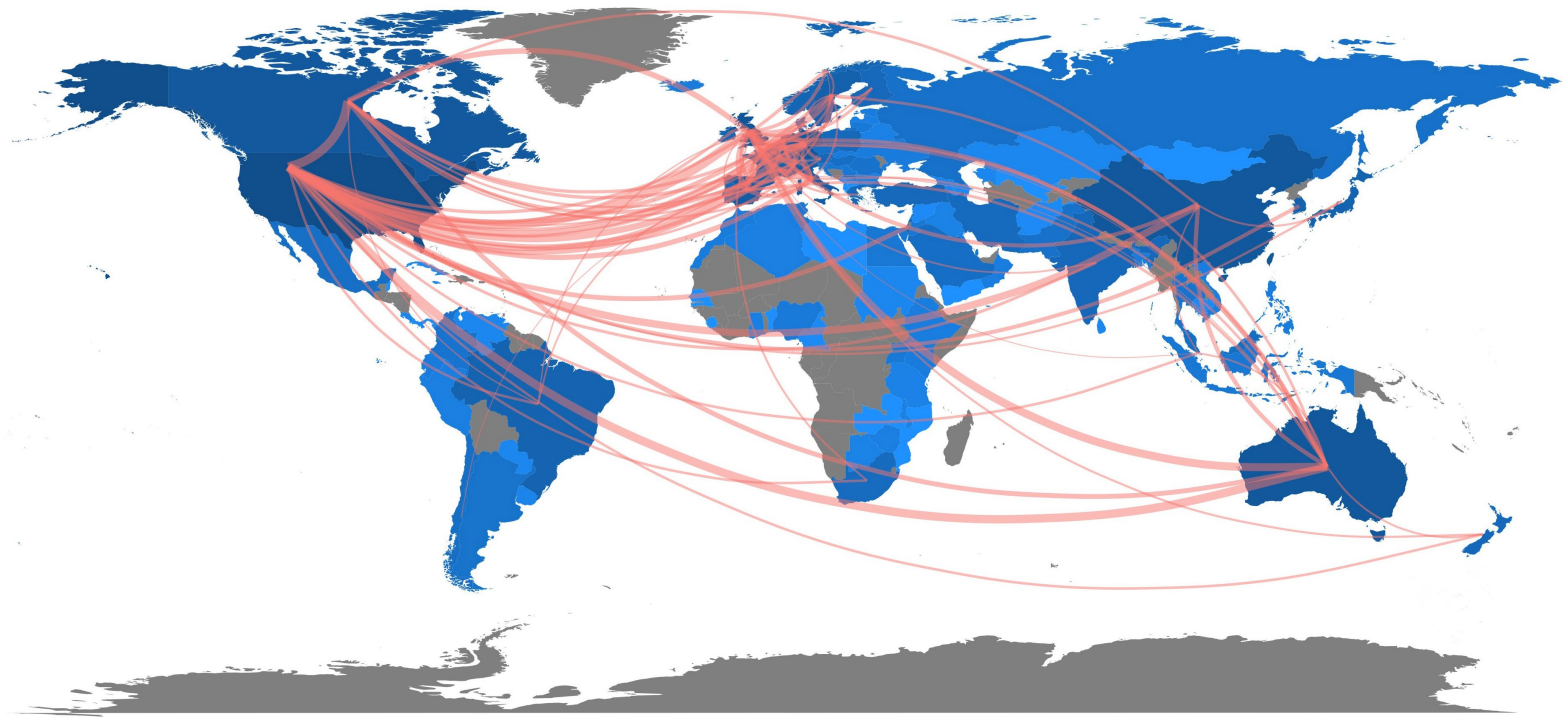
International cooperation between countries.

Over 110 countries made contributions to the research of digital cognitive tests. Table 1 shows the top 10 countries and institutions with the largest contribution. The United States ranked first with a total of 3,953 publications, followed by China (1,132), the United Kingdom (1,129), Australia (830), Germany (764) and Canada (735). Publications from these countries account for more than 75% of the total output. International cooperation between countries is shown in Figure 3. The United States was the most active country. The most frequent cooperation was between the United States and the United Kingdom (frequency = 335), followed by the cooperation between the USA and Canada (frequency = 306), and between the United States and Australia (frequency = 265). As is depicted in the Figure 3, most of the research collaborations occurred among the countries in North America, European, East Asia and Australia.

**Table 1:** Top 10 countries/regions and institutions for publication.

Country ranking			Institutional ranking			
Rank	Country	Articles	Rank	Affiliation	Articles	Country
1	United States	3,953	1	University of Toronto	450	Canada
2	China	1,132	2	University of Pittsburgh	318	United States
3	United Kingdom	1,129	3	University of Melbourne	315	Australia
4	Australia	830	4	University of Pennsylvania	312	United States
5	Germany	764	5	King’s College London	310	United Kingdom
6	Canada	735	6	University of Michigan	300	United States
7	Netherlands	469	7	Karolinska Institutet	277	Sweden
8	Italy	433	8	University of Sydney	270	Australia
9	Spain	412	9	Harvard Medical School	269	United States
10	Korea	261	10	University of California San Francisco	268	United States

University of Toronto (450 papers) was the institution that published the most productions, followed by University of Pittsburgh (318 papers), University of Melbourne (315 papers), University of Pennsylvania (312 papers), King’s College London (310 papers) and University of Michigan (300 papers) (Table 1). Of the top 10 most productive institutions, 5 were located in the United States (University of Pittsburgh, University of Pennsylvania, University of Michigan, Harvard Medical School and University of California San Francisco), indicating that institutions from the United States were more actively involved in the study of cognitive assessment.

#### 4.1.3. Contributions of authors

**Figure 4:**
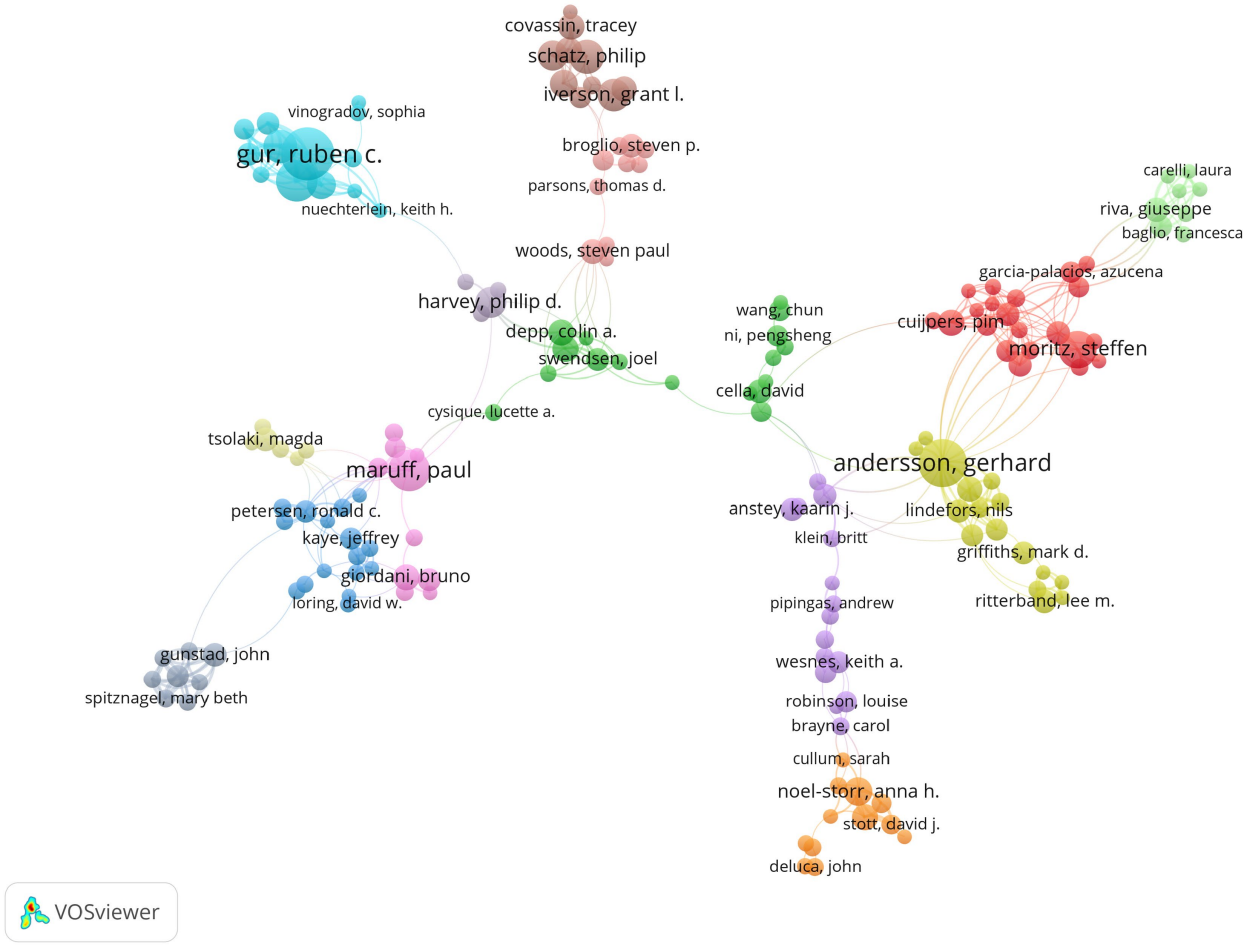
Cooperation between researchers from various institutions.

More than 55,000 authors contributed to the studies on digital testing tools. The contributions of the top 10 authors are shown in Table 2. Ruben C. Gur from University of Pennsylvania contributed the most in this field, with a total of 71 articles (H-index = 25). Gerhard Andersson ranked first in terms of authors’ local impact with an H-index of 29. Figure 4 shows the cooperation between researchers from various institutions. The minimum number of documents per author was set as 8, and 166 authors were included in the analysis. There were 14 clusters, but the links between different clusters were relatively sparse, which revealed the lack of full cooperation or communication between research teams or labs in this area.

**Table 2:** Top 10 authors with the most publications.

Authors	Institution	Articles	H-index
(GUR RC) Ruben C. Gur	University of Pennsylvania, United States	71	25
(ANDERSSON G) Gerhard Andersson	Linköping University, Sweden	56	29
(MARUFF P) Paul Maruff	Monash University, Australia.	53	27
(GUR RE) Raquel E Gur	University of Pennsylvania, United States	46	21
(MORITZ S) Steffen Moritz	University Medical Center Hamburg-Eppendorf, Germany	37	15
(SCHATZ P) Philip Schatz	Saint Joseph’s University, United States	34	20
(LEE J) Joohee Lee	University of Ulsan College of Medicine, Korea	32	13
(LEE S) Seongwon Lee	Ajou University School of Medicine, Korea	32	10
(MOORE TM) Tyler M Moore	University of Pennsylvania, United States	32	12
(IVERSON GL) Grant L Iverson	Harvard Medical School, United States	30	18

H-index: the Hirsch index. The h-index stands for a count of the largest number of papers (h) from a journal or author that have at least (h) number of citations.

#### 4.1.4. Analysis of the journals and articles

The articles on digital cognitive assessment were published across a wide range of 2,666 journals. These jurnals were inclusive multidisciplinary journals, or professional journals classified into psychology, neurology and so on. Additionally, most articles were published in open access journals. Table 3 lists the top 15 journals by the number of articles published. Journals published in the way of open-access tend to publish more articles and obtain a higher number of total citations than non–open access journals (12).

**Table 3:** Top 15 journals with the most publications.

	Sources	Articles	IF	JCR-c	Publisher	H-index	TC
1	Frontiers in Psychology	276	4.232	Q1	Frontiers	31	4,308
2	Plos One	234	3.752	Q2	PLOS	43	6,313
3	BMJ Open	176	3.006	Q2	BMJ Publishing Group	22	2,255
4	Journal of Medical Internet Research	170	7.076	Q1	JMIR Publications Inc.	39	4,865
5	Cochrane Database of Systematic Reviews	140	12.008	Q1	Wiley	49	8,296
6	International Journal of Environmental Research and Public Health	137	4.614	Q1	MDPI	14	1,231
7	Frontiers in Psychiatry	117	5.435	Q2	Frontiers	15	946
8	Journal of Alzheimers Disease	115	4.16	Q2	IOS Press	24	1,841
9	Archives of Clinical Neuropsychology	107	3.448	Q2	Oxford University Press (OUP)	30	3,587
10	Computers in Human Behavior	99	8.957	Q1	Elsevier	34	3,510
11	Journal of Affective Disorders	88	6.533	Q1	Elsevier	23	2,015
12	Trials	87	2.728	Q4	BioMed Central (BMC)	16	804
13	Journal of Clinical and Experimental Neuropsychology	82	2.283	Q3	Taylor and Francis	23	2,657
14	Frontiers in Aging Neuroscience	78	5.702	Q1	Frontiers	17	1,111
15	Journal of the International Neuropsychological Society	78	3.114	Q2	Cambridge University Press	26	2,296

IF: impact factor (2020–2021).

JCR-c: Journal Citation Reports category (2021).

H-index: the Hirsch index. The h-index stands for a count of the largest number of papers (h) from a journal or author that have at least (h) number of citations.

TC, Total Citation, the number of times each manuscript has been cited.

Table 4 displays the 15 most cited articles from the total of 13,244 documents in this study. They were published from 2003 to 2015. The citations of recently published studies were low compared with those of earlier publications. This may lead to the underestimation of the importance of the new publications (40). Of these articles, the top two articles were clinical studies to test the validity of two computerized neurocognitive test batteries, CNS Vital Signs (CNSVS) (41) and CogState (42). Two reviews about assessing or detecting cognitive changes employing computerized testing in the elderly were also highly cited in this field (43, 44), indicating that using digital technology for early detection of changes in cognition in the aging population was the concern of academia. Apart from this, five of these 15 high-cited articles were relevant to sport-related concussion and cognition assessment methods (45–49). Digital neuropsychological testing in the management of sport-related concussions was also a hot topic.

**Table 4:** The top 15 cited articles related to digital cognitive assessment research from 2003 to 2023.

	Title	First Author	Journal	Year	LC	GC
1	Reliability and validity of a computerized neurocognitive test battery, CNS Vital Signs	C. Thomas Gualtieri	Archives of Clinical Neuropsychology	2006	153	519
2	Validity of the CogState brief battery: relationship to standardized tests and sensitivity to cognitive impairment in mild traumatic brain injury, schizophrenia, and AIDS dementia complex	Paul Maruff	Archives of Clinical Neuropsychology	2009	124	391
3	Status of computerized cognitive testing in aging: a systematic review	Katherine Wild	Alzheimers & Dementia	2008	118	272
4	Test–retest reliability of computerized concussion assessment programs	Steven P Broglio	Journal of Athletic Training	2007	103	194
5	Practice effects associated with the repeated assessment of cognitive function using the CogState battery at 10-min, one week and one month test–retest intervals	Marina G Falleti	Journal of Clinical and Experimental Neuropsychology	2006	88	283
6	Long-term test–retest reliability of baseline cognitive assessments using ImPACT	Philip Schatz	The American Journal of Sports Medicine	2010	82	167
7	One-year test–retest reliability of the online version of ImPACT in high school athletes	R J Elbin	The American Journal of Sports Medicine	2011	78	138
8	The effects of practice on the cognitive test performance of neurologically normal individuals assessed at brief test–retest intervals	Alexander Collie	Journal of the International Neuropsychological Society	2003	77	316
9	CogSport: reliability and correlation with conventional cognitive tests used in postconcussion medical evaluations	Alexander Collie	Clinical Journal of Sport Medicine	2003	77	212
10	A cognitive neuroscience-based computerized battery for efficient measurement of individual differences: standardization and initial construct validation	Ruben C Gur	Journal of Neuroscience Methods	2010	77	327
11	Is neuropsychological testing useful in the management of sport-related concussion?	Christopher Randolph	Journal of Athletic Training	2005	72	195
12	Computerized cognitive testing for older adults: a review	Stelios Zygouris	American Journal of Alzheimer’s Disease and other Dementias	2015	72	154
13	A meta-analysis of cognitive remediation for schizophrenia: methodology and effect sizes	Til Wykes	American Journal of Psychiatry	2011	68	1,076
14	The “value added” of neurocognitive testing after sports-related concussion	Derk A Van Kampen	The American Journal of Sports Medicine	2006	65	228
15	Validity of ImPACT for Measuring Processing Speed Following Sports-Related Concussion	Grant L. Iverson	Journal of Clinical and Experimental Neuropsychology	2005	63	189

LC, Local Citations; GC, Global Citations.

### 4.2. Overview of research trends and hotspots

#### 4.2.1. High-frequency keywords and cluster analysis

**Figure 5:**
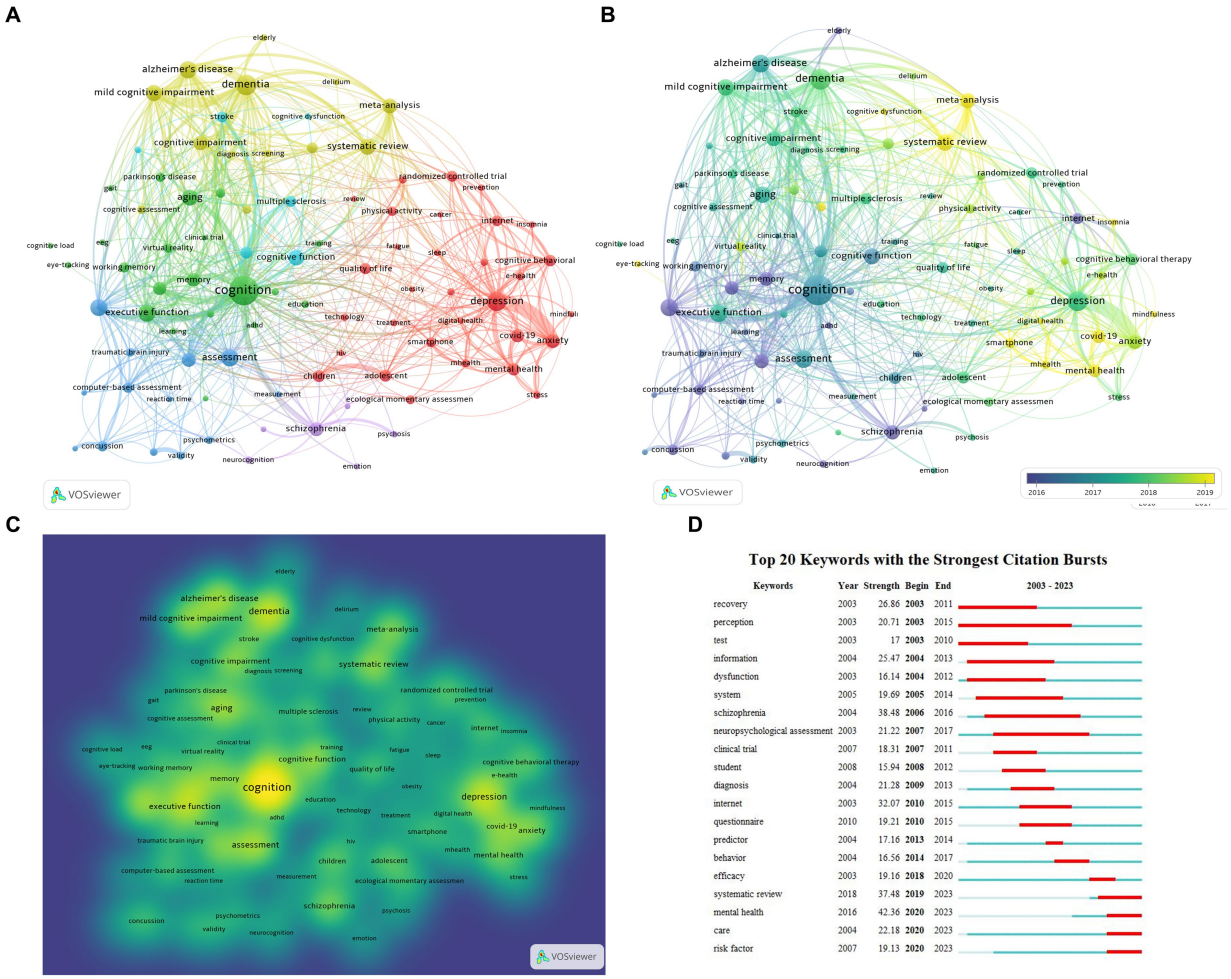
Analysis of co-occurring author keywords about digital cognitive assessment. **(A)** Network visualization of author keywords. The size of the label and the circle of an item is determined by the occurrence frequency of keywords. The color of an item is determined by the cluster to which the item belongs. **(B)** Overlay visualization of author keywords. The color of an item is determined by the time of appearance. **(C)** An item density visualization map of author keywords. The color of a point is closer to yellow when there are more keywords with higher weights in its neighborhood, and it is closer to blue when there are fewer keywords with lower weights in its neighborhood. **(D)** Visualization map of top 20 keywords with the strongest citation bursts.

A total of 23,299 keywords were extracted from the 13,244 articles. Following a previous study (40), keywords with similar meanings were merged and keywords with general meaning were filtered out manually. Only keywords with a minimum of occurrences as 60 were visualized, and at last, 90 keywords met this threshold and were visualized by Vosview (Figures 5A–C). The occurrence frequency of keywords was presented by the size of nodes, while the strength between two words was presented by the distance between two nodes (Figure 5A). The most frequently used keywords were “cognition,” “dementia,” “depression,” “assessment,” “executive function” “Alzheimer’s disease,” and “mild cognitive impairment” and so on (Table 5 and Figure 5A). The color of each circle indicates which cluster it belongs to. Keywords with higher correlations were classified into the same cluster with the same color, which roughly reflected the focus of recent research (40). In this study, selected keywords were roughly divided into five clusters. Cluster 1 is colored in red, with the main keywords focusing on mental health and digital assessment during the COVID-19 pandemic, including terms like “depression,” “anxiety,” “COVID-19,” “digital health” and “e-health.” Cluster 2 in green focuses on different cognition domains, such as “attention,” “executive function,” “memory” and “working memory.” Cluster 3 in dark blue color focuses on cognitive assessment, like “reliability” and “assessment.” Cluster 4 in yellow color focuses on cognitive diseases, including “dementia,” “Alzheimer’s disease” and “mild cognitive impairment.” Cluster 5 in purple color focuses on psychosis with the main keywords “psychosis,” “bipolar disorder” and “schizophrenia.” Cluster 6 in light blue color emphasizes cognitive dysfunction and rehabilitation. Figure 5B displays the overlay visualization of author keywords, in which the terms in blue color appeared earlier and the terms in yellow color appeared recently. Keywords, such as “concussion,” “memory,” “assessment” and “cognition” were the past major topics, while the topics about “mental health,” “depression,” “meta-analysis,” “COVID-19,” “digital health,” and “smartphone” have been popular in recent years. Figure 5C is a density visualization map of included keywords using VOSviewer. The color of a point is closer to yellow when the keyword has a higher degree of attention, and conversely, it is closer to blue. “Cognition,” “dementia,” “depression” have gain the most attention.

**Table 5:** The top 20 keywords.

	Keyword	Occurrences
1	Cognition	939
2	Dementia	511
2	Depression	465
3	Assessment	383
4	Alzheimer’s disease	380
5	Executive function	372
6	Neuropsychological test	363
7	Mild cognitive impairment	335
8	Systematic review	331
9	Aging	329
10	Cognitive function	314
11	Meta-analysis	294
12	Cognitive impairment	278
13	Anxiety	272
14	Schizophrenia	270
15	Neuropsychology	269
16	Memory	257
17	Attention	244
18	COVID-19	242
19	Mental health	219
20	Adolescent	208

**Figure 6:**
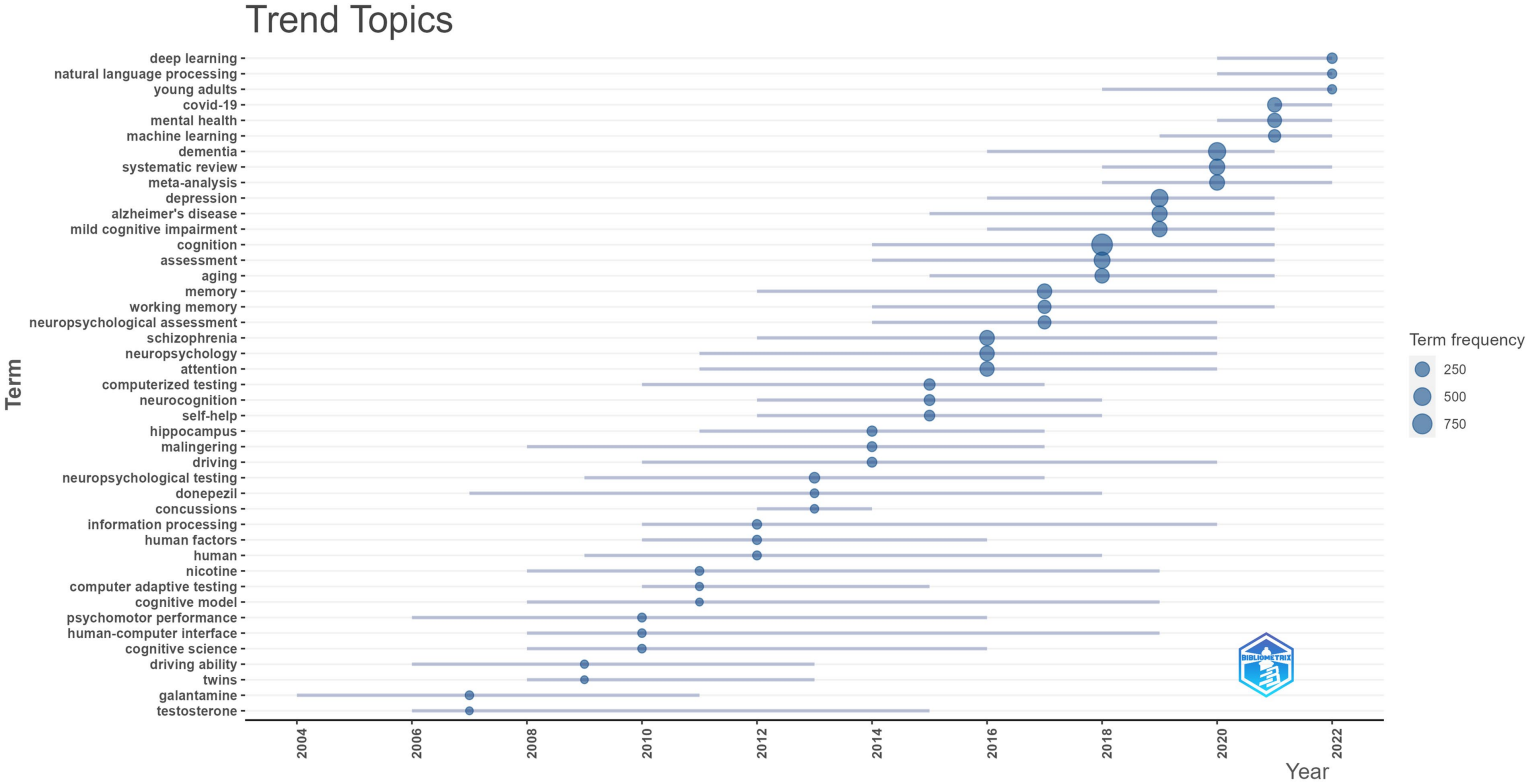
Annual topic trend presented by the Bibliometrix. In 2016, the research on machine learning was started and lasted until 2021. From 2020, the work on deep learning and natural language processing arose.

Burst keyword detection was performed in the Citespace software in order to identify the emerging concepts cited frequently (38). Burst keyword detection identifies abrupt fluctuations in the frequency or occurrence of particular keywords or phrases during a certain period (50). Figure 5D is a visualization map of the top 20 keywords with the strongest citation bursts from 2003 to 2023. Over the past two decades, mental health ranked first with the highest burst strength (42.36), followed by schizophrenia (38.48), systematic review (37.48) and Internet (32.07). Mental health, care and risk factor burst from 2020 to 2023, which may be the current research hotspots. According to the results of the “Trend Topic” analysis in the Bibliometrix, terms including “deep learning,” “natural language processing,” and “machine learning,” were also the “hot words” in this area (Figure 6). In 2016, the research on machine learning was started and lasted until 2021. From 2020, the work on deep learning and natural language processing arose. This means that there has been research on the application of artificial intelligence technology to the measurement of cognitive ability in recent years.

#### 4.2.2. Analysis of references

**Figure 7:**
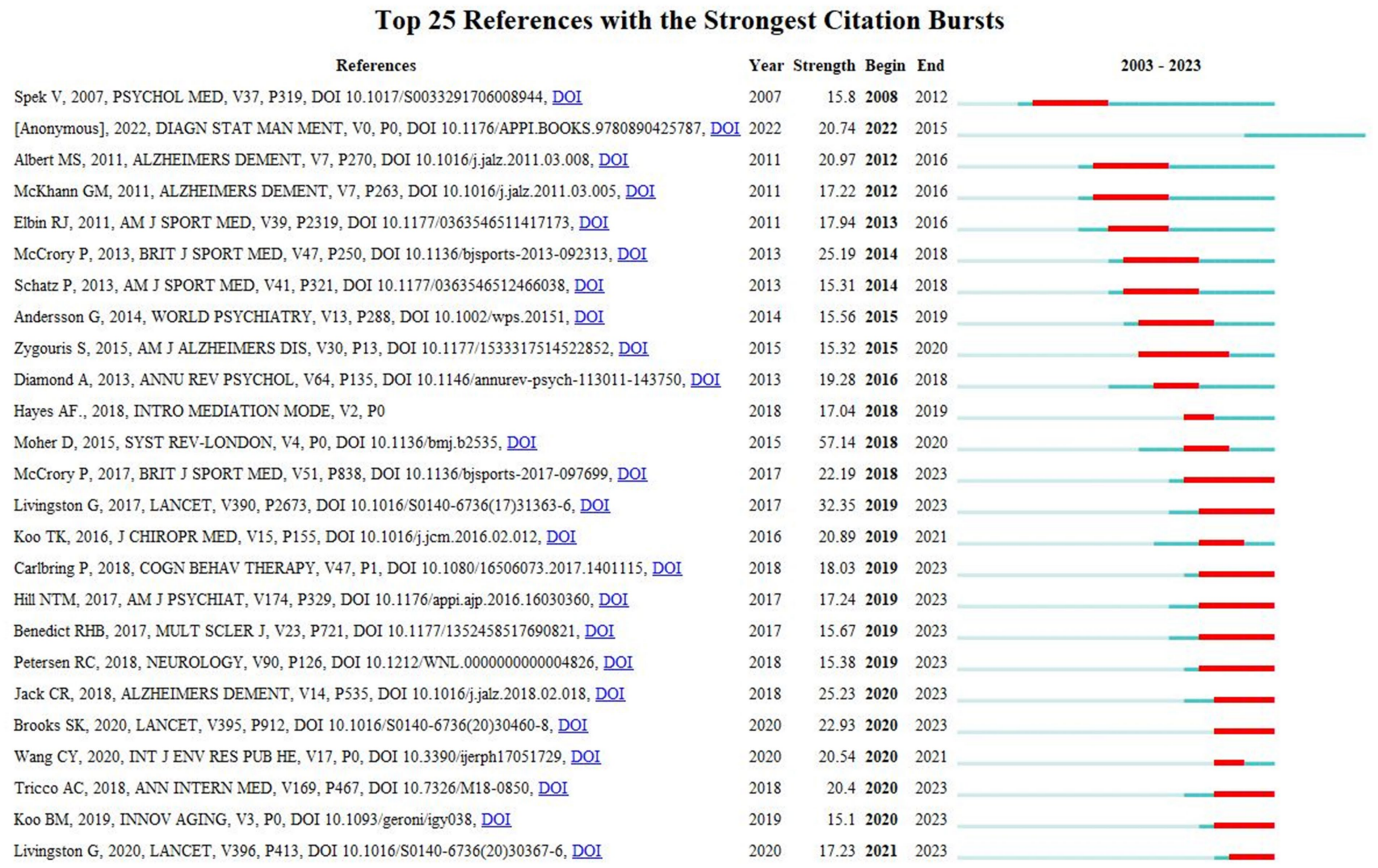
Top 25 references with the highest citation bursts. The minimum duration of the burst was 2 years, while the blue line represents the observed time interval from 2003 to 2023 and the red line represents the burst duration.

Reference citation burst detection usually revealed a work of great potential or interest and hit a key part of the complex system in the academic field (38). The 10 most cited references are shown in Table 6, and Figure 7 illustrates the top 25 references with the highest citation burst. The minimum duration of the burst was 2 years, while the blue line represents the observed time interval from 2003 to 2023 and the red line represents the burst duration. Of these articles, the methodological article entitled “Preferred reporting items for systematic reviews and meta-analyzes: the PRISMA statement” written by David Moher has the highest burst strength (57.14). Moreover, the citation burst for several papers is still ongoing, such as Carlbring et al. (51), Cogn Behav Therapy (18.03), Jack et al. (52), Alzheimers Dement (25.23), Koo et al. (7), Innov Aging (15.1) and Livingston et al. (53), Lancet (17.23). These papers covered the academic fields of meta-analysis, cognitive behavior therapy, the diagnosis of Alzheimer’s disease or milf cognitive impairment, dementia prevention and intervention, which suggests that such research topics are likely to remain popular in the future and may become potential frontiers in the research field of cognitive assessment.

**Table 6:** Top 10 cited references related to cognitive assessment.

	Author (Year)	Title	Journal	Frequency
1	Preacher K. J. (2004)	SPSS and SAS procedures for estimating indirect effects in simple mediation models	Behavior Research Methods, Instruments, & Computers	10,983
2	Rouder J. N. (2009)	Bayesian t tests for accepting and rejecting the null hypothesis	Psychonomic Bulletin & Review	2,354
3	Richardson M. (2012)	Psychological correlates of university students’ academic performance: a systematic review and meta-analysis	Psychological Bulletin	1,611
4	Ngandu, T. (2015)	A 2 year multidomain intervention of diet, exercise, cognitive training, and vascular risk monitoring versus control to prevent cognitive decline in at-risk elderly people (FINGER): a randomized controlled trial	Lancet	1,602
5	Dienes Z. (2014)	Using Bayes to get the most out of non-significant results	Frontiers in Psychology	1,192
6	Pilkonis P. A. (2011)	Item Banks for Measuring Emotional Distress From the Patient-Reported Outcomes Measurement Information System (PROMIS®): Depression, Anxiety, and Anger	Assessment	1,117
7	Wykes T. (2011)	A meta-analysis of cognitive remediation for schizophrenia: methodology and effect sizes	American Journal of Psychiatry	1,076
8	Heyn P. (2004)	The effects of exercise training on elderly persons with cognitive impairment and dementia: A meta-analysis	Archives of Physical Medicine and Rehabilitation	814
9	Green C. R. (2007)	Executive function deficits in children with fetal alcohol spectrum disorders (FASD) measured using the Cambridge Neuropsychological Tests Automated Battery (CANTAB)	Journal of child psychology and psychiatry	797
10	Sonuga-Barke E. J. (2013)	Nonpharmacological interventions for ADHD: systematic review and meta-analyzes of randomized controlled trials of dietary and psychological treatments	American Journal of Psychiatry	674

**Figure 8:**
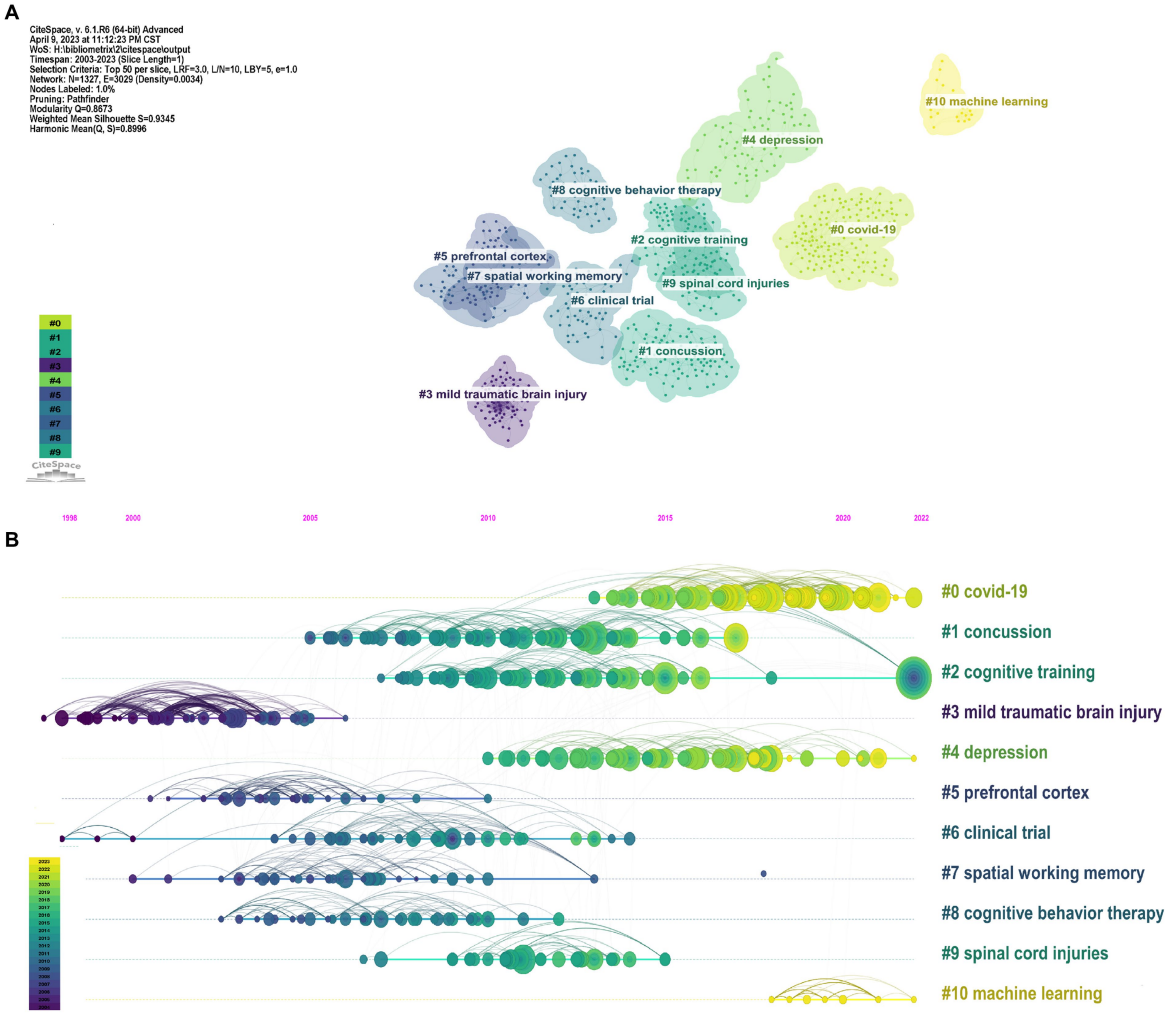
Co-citation references network and correspondent clustering analysis obtained with CiteSpace. **(A)** Network visualization of the results of the cluster analysis of highly co-cited references in the field of digital cognitive assessment. **(B)** Timeline diagram of cluster analysis in panel A.

Co-cited references, simultaneously cited by other 2 publications, represent the scientific relevance of publications (54). Document co-citation analysis, the most representative analysis function of CiteSpace, can evaluate the correlation between documents. By analyzing the co-citations of cited references, the background and knowledge base of digital cognitive assessment can be discovered. Clusters were constructed based on keywords extracted from references using log-likelihood ratio through CiteSpace. After clustering of co-cited references, 1,327 nodes, 3,028 edges, and 238 main clusters were acquired by the log-likelihood ratio algorithm in the CiteSpace software. We presented the 11 clusters (Figure 8A) and their timelines for each cluster label (Figure 8B). The 11 largest clusters were “covid-19 (Cluster #0, size = 146, Silhouette = 0.915),” “concussion (Cluster #1, size = 113, Silhouette = 0.950),” “cognitive training (Cluster #2, size = 97, Silhouette = 0.901),” “mild traumatic brain injury (Cluster #3, size = 88, Silhouette = 0.936),” “depression (Cluster #4, size = 85, Silhouette = 0.959),” “prefrontal cortex (Cluster #5, size = 57, Silhouette = 0.918),” “clinical trial (Cluster #6, size = 56, Silhouette = 0.879),” “spatial working memory (Cluster #7, size = 55, Silhouette = 0.919),” “cognitive behavior therapy (Cluster #8, size = 48, Silhouette = 0.945),” “spinal cord injuries (Cluster #9, size = 38, Silhouette = 0.956),” and “machine learning (Cluster #10, size = 26, Silhouette = 1).” According to the results, the cluster structure was significant and highly reliable, with a total modularity Q of 0.8673 and a weighted mean Silhouette of 0.9345. The position of the nodes in these clusters suggests the pioneering research. For example, clusters #1, #3 and #4 study the clinical application of cognitive asssessment. Clusters #2 and #8 study the therapy. Cluster #0 and #10 are related to popular topics in recent years. The position of the nodes in these clusters suggests the pioneering research focus.

## 5. Discussion

### 5.1. Main findings

We conducted a bibliometric analysis, providing a thorough overview of international research on digital cognitive assessment from 2003 to 2023. Our study contained more than 13 thousand articles from 55,490 authors published in 2,666 journals and 480,837 references. Interest in this topic has grown rapidly over the preceding two decades. The findings show that a total of 13,244 articles have been published. The trend substantially increased in recent years, and it is estimated that this growth will continue in the next few years. This serves as a reminder that electronic cognitive assessment tools are expected to be widely used in clinical situations. This trend is also consistent with the widespread use of digital devices in other clinical settings (55–57). In terms of literature output, the United States was a highly productive country in this field. China, the United Kingdom, Germany, Canada and Australia have a dominant position in this field as well. Half of the 10 most productive institutions and authors were from the United States, indicating that the United States was the leading country in this field.

### 5.2. Collaboration relationship among countries/regions and authors

The United States is not only the most productive country, but also serves as the hub of global trade. In the area of digital cognitive evaluation, close research collaboration has increased among the top 10 most productive countries. The United States established strong research collaboration with European countries. Moreover, most of the research collaborations occurred among the countries in North America, European, East Asia and Australia. Academic capability, to a large extent, depends on the governmental economic status and its expenditure on healthcare (58). The majority of these countries are economic powers with strong scientific and technological capabilities. They are the global leaders in electronic information science and technology, making it possible for them to conduct research in this field.

Another potential incentive for their cooperation may be the reality of an aging population they had to cope with (59). According to the *World Population Prospects 2022* unveiled by the United Nations (60), Europe and Northern America had the largest proportion of the older population, followed by Australia. What’s more, one in every four persons living in Europe and Northern America could be aged 65 or over (60). Elderly people frequently have dementia and other forms of cognitive impairment since becoming older is still the biggest risk factor for dementia (61). In the following decades, dementia sufferers will become more prevalent as the population ages (62). In an aging population, dementia prevention is a public health issue that cannot be disregarded (53). There is a great demand for more accurate, convenient and fast cognitive measurement tools. That may explain why researchers in these countries put so much effort into studies assessing cognitive ability.

At the same time, it should be noted that, apart from China, there are fewer studies from developing countries, and less national collaboration between developing countries and developed countries. Another advantage of digitized cognitive assessment tools is the lower requirement for extra space and professional personnel, allowing remote evaluation, self-assessment or self-management outside of health care (7). This demonstrates how useful electronic instruments might be in rural or underdeveloped areas, particularly where there are few medical resources and a high population density (63). Therefore, the cooperation between developed and developing countries is expected to become closer in the future.

The collaborative relationship between the authors was also a focus of this study. The analysis about co-authorship between researchers is helpful to identify existing partnerships and explore potential collaborators (58). Similar to the findings about regional distribution, several institutions and authors from North America, Western Europe, East Asia and Australia published widely on this topic. The results show that there is a certain degree of cooperative networks among researchers in this field, but most of this cooperation is concentrated within a few groups or teams. Since there are not numerous links between different academic institutions or groups, more chances for researchers from various fields to work together should be offered.

### 5.3. The new trends

Finding hotspots is a crucial component of bibliometric analysis, which foretells potential study ideas. Future investigations are always guided by the existing hotspots and trends. The essential idea and content of one article are reflected in keywords, which are highly compressed and generalized words. Our findings showed that concussion was a hot topic in the past in this field. These findings are confirmed by the fact that many of the established digital tools were originally designed to assess mild traumatic brain injury or concussion in military and sports psychology (16). But now, the most frequently used keywords focused on terms related to cognitive function and cognitive disorders, including “Alzheimer’s disease” and “mild cognitive impairment.” This demonstrates that digital tools are applied to diverse clinical groups including neurocognitive disorders. Disease diagnosis and severity evaluation are the principal uses of digital cognitive assessment tools nowadays. Apart from this, other frequently used keywords focused on terms related to mood disorders, like depression and anxiety, and appeared with the keyword “COVID-19.” The burst detection analysis about keywords also implied that mental health care was actively discussed in the past few years. This reflects the increased attention that researchers are paying to mental health during the COVID-19 pandemic (64–66). Previous studies indicated that the COVID-19 pandemic had contributed to over 25% increase in the prevalence of anxiety and depression globally (66). Another observational study found a relatively high frequency of cognitive impairment in executive functioning, processing speed and memory encoding among hospitalized patients who had contracted COVID-19 several months before (67). Due to the social isolation brought on by the unprecedented pandemic, traditional face-to-face cognitive assessment has grown to be very challenging. However, this public health emergency has triggered a rapid shift in the way of health care, boosting the global adoption and usage of telehealth or other electronic solutions (68, 69). This offered a critical opportunity for the popularity of electronic cognitive assessment tools (70–72). In the post-pandemic era, digital health approaches will continue to provide services for patients in neurological, psychiatric and mental health care (73).

In the part of analyzing the references, we identified several clusters using the co-occurrence clustering function, with each cluster representing a main theme or topic. Some references with high citations were clustered in the domains of machine learning. Besides, according to the results from the “Bibliometrix” package, “machine learning,” “deep learning” and “natural language processing” were also the main trend topics in the last 4 years. In recent years, numerous complicated medical problems have been solved using machine learning (ML) and other artificial intelligence (AI) techniques (74). ML builds prediction models with high accuracy, and thus enhances the diagnostic performance of many diseases, mainly in cancer, medical imaging and wearable sensors (74). Deep learning, a branch of ML, has quickly become the method of choice for assessing brain imaging, like functional magnetic resonance imaging (fMRI) and electroencephalogram (EEG) (74). At a cognitive and behavioral level, ML systems can extract reliable features originating from the neuropsychological assessment, automatically classify different disease phenotypes, identify development stages, and even predict disease conversion (75, 76). With the emergence of studies on the utilization of ML algorithms to neuropsychological tests and the ever-upgrading algorithms (75, 77), electronic tools that integrate detection, diagnosis, cognitive training and therapy would be a promising direction in clinical practice.

### 5.4. Limitation

In this study, we provide a comprehensive picture of the basic research information in the field of computerized cognitive assessment. The emergence of digital technologies has opened up a new era for cognitive testing tools. Although some discoveries can be found in our analysis, there are still several inevitable limitations. Similar to the majority of bibliometrics articles, data were solely collected from the English articles or reviews included in WoSCC databases. This is partly due to the limitations of scientometric software (37), because it is extremely difficult to directly merge the data from two different databases, such as Scopus and Embase (40). So some excellent publications might have been missed, like gray articles, meeting abstracts and patent materials.

## 6. Conclusion

This bibliometric analysis presented the overall structural framework and identified the key perspectives of the research on digital cognitive assessment. In conclusion, research into this field is accelerating rapidly, receiving growing attention in the past decades. Assisting cognitive assessment in mental health disorders using computer-assisted tools is the current hotspot. Researchers and institutions from America are the top contributors to this topic. America, Canada and other high-income countries or regions represent the main force regarding this domain. Digital evaluation technology is on the ascendant. Our findings will assist researchers and policymakers in grasping the basic research status and formulating the plan for the future.

## Data availability statement

The original contributions presented in the study are included in the article/Supplementary material, further inquiries can be directed to the corresponding author/s.

## Author contributions

LC designed this study, performed the statistical analysis, and drafted the manuscript. WZ and DP revised the manuscript. All authors contributed to the article and approved the submitted version.

## Funding

This research was funded by the Central Health Research Project (grant number 2020ZD10), and the National Key R&D Program of China (grant number 2022YFC2010103).

## Conflict of interest

The authors declare that the research was conducted in the absence of any commercial or financial relationships that could be construed as a potential conflict of interest.

## Publisher’s note

All claims expressed in this article are solely those of the authors and do not necessarily represent those of their affiliated organizations, or those of the publisher, the editors and the reviewers. Any product that may be evaluated in this article, or claim that may be made by its manufacturer, is not guaranteed or endorsed by the publisher.
