# Supplementary material for: Research on digital tool in cognitive assessment: a bibliometric analysis
Source: Front Psychiatry. 2023 Aug 23;14:1227261. doi: 10.3389/fpsyt.2023.1227261 (PMC10482043; doi:10.3389/fpsyt.2023.1227261)
Supplement: Supplementary file 1 [file Data_Sheet_1.docx]

**Supplementary Appendix**

[1. Search Strategy 3](#_Toc134171339)

[2. The steps of literature identification and screening process. 4](#_Toc134171340)

[3. The main information of the data 5](#_Toc134171341)

[4. Annual publications 6](#_Toc134171342)

[5. Trends topics 7](#_Toc134171343)

[6. Top 10 cited references related to cognitive assessment 8](#_Toc134171344)

[7. Top 25 references with the strongest citation bursts 9](#_Toc134171345)

[8. Three-field plot of the Author Keywords analysis 10](#_Toc134171346)

# 1. Search Strategy

# Web of Science Search Strategy (v0.1)

# Database: Web of Science Core Collection

# Entitlements:

- WOS.IC: 1993 to 2023

- WOS.CCR: 1985 to 2023

- WOS.SCI: 1900 to 2023

- WOS.AHCI: 1975 to 2023

- WOS.ESCI: 2018 to 2023

- WOS.ISTP: 1997 to 2023

- WOS.SSCI: 1900 to 2023

- WOS.ISSHP: 1999 to 2023

# Searches:

1: TS=(digital) OR TS=(computerized) OR TS=(electronic) OR TS=(online) OR TS=(smartphone) OR TS=(mobile) OR TS=(tablet) OR TS=(computer) OR TS=(Internet ) OR TS=(web) Editions: WOS.SCI,WOS.SSCI Date Run: Wed Apr 05 2023 14:48:52 GMT+0800 (China Standard Time) Results: 2946025

2: TS= (neuropsychological tests) OR TS= (neurocognitive test) OR TS= (psychological tests) OR TS= (mental status and dementia tests) OR TS= (cognitive tests) OR TS= (cognitive assessment) OR TS= (cogniti* assessment) OR TS= (cogniti* tests) Editions: WOS.SCI,WOS.SSCI Date Run: Wed Apr 05 2023 14:50:55 GMT+0800 (China Standard Time) Results: 146629

3: #2 AND #1 Editions: WOS.SCI,WOS.SSCI Date Run: Wed Apr 05 2023 14:51:04 GMT+0800 (China Standard Time) Results: 14844

4: #3 and Article or Review Article (Document Types) Editions: WOS.SCI,WOS.SSCI Timespan: 2003-01-01 to 2023-04-03 Date Run: Wed Apr 05 2023 14:56:38 GMT+0800 (China Standard Time) Results: 13698

5: #3 and Article or Review Article (Document Types) and English (Languages) Editions: WOS.SCI,WOS.SSCI Timespan: 2003-01-01 to 2023-04-03 Date Run: Wed Apr 05 2023 14:56:50 GMT+0800 (China Standard Time) Results: 13470

# 2. The steps of literature identification and screening process.


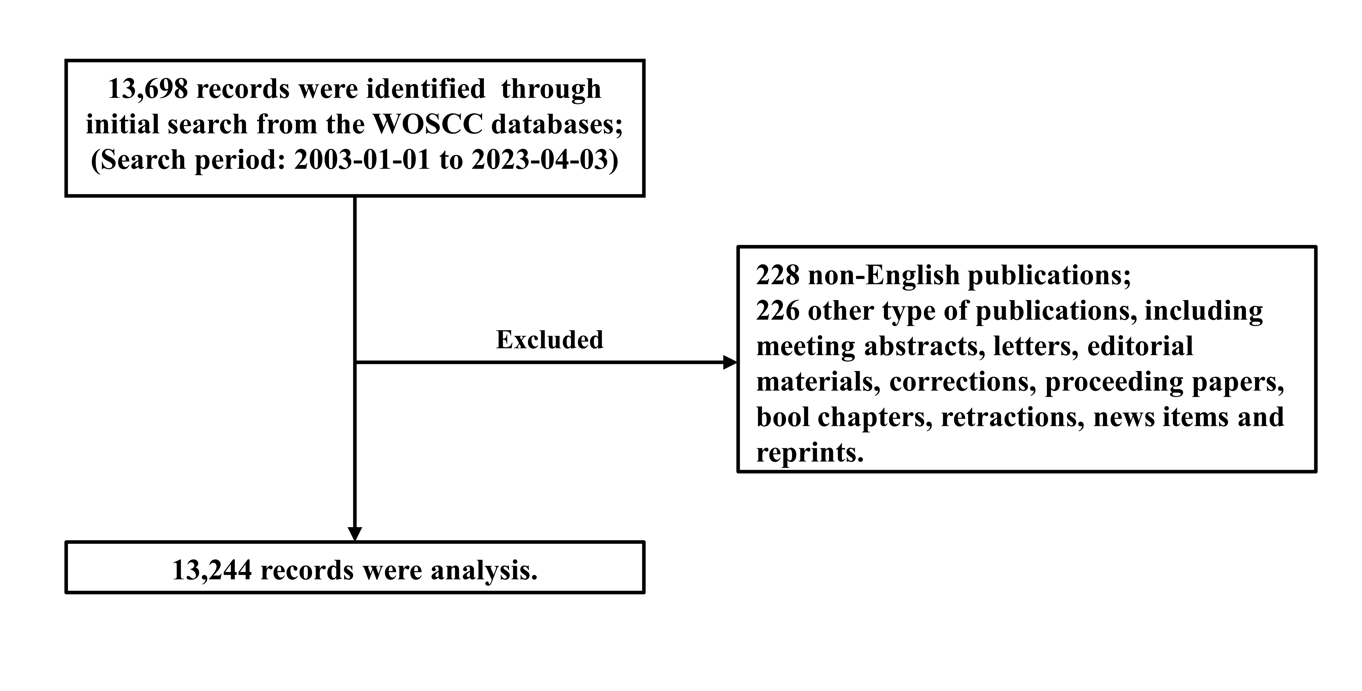


# 3. The main information of the data

**Table S1. The main information**

| **Description** | **Results** |
| --- | --- |
| **MAIN INFORMATION ABOUT DATA** |  |
| Timespan | 2003:2023 |
| Sources (Journals, Books, etc) | 2666 |
| Documents | 13244 |
| Annual Growth Rate % | 5.51 |
| Document Average Age | 6.11 |
| Average citations per doc | 28.19 |
| References | 480837 |
| **DOCUMENT CONTENT**S |  |
| Keywords Plus (ID) | 18732 |
| Author's Keywords (DE) | 23006 |
| AUTHORS |  |
| Authors | 55490 |
| Authors of single-authored docs | 460 |
| **AUTHORS COLLABORATION** |  |
| Single-authored docs | 487 |
| Co-Authors per Doc | 5.84 |
| International co-authorships % | 27.69 |
| **DOCUMENT TYPES** |  |
| article | 10733 |
| article; early access | 312 |
| review | 2124 |
| review; early access | 75 |

# 4. Annual publications

**Figure S1. The number of annual publications**

# 5. Three-field plot of the Author Keywords analysis

**Figure S2**. **Three-field plot of the Author Keywords analysis**


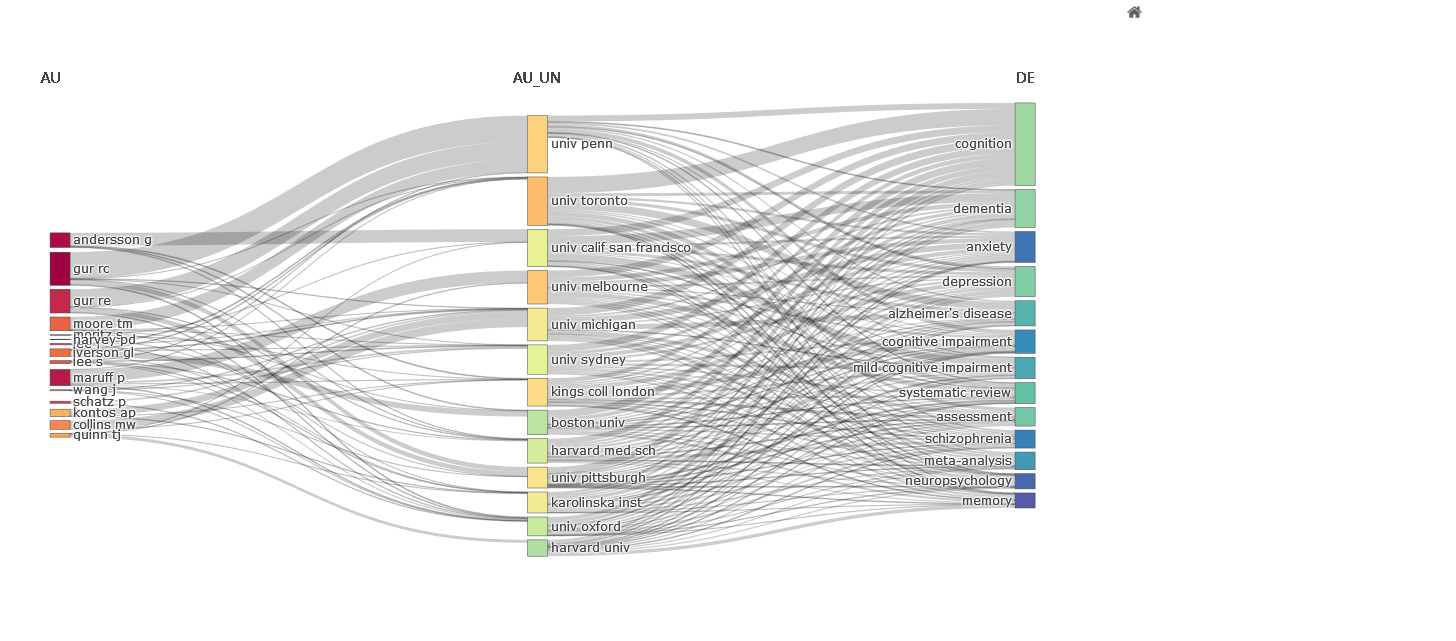


**Notes: Three-field plot of the Author Keywords analysis**

Visualize the main items of three fields (e.g. authors, affiliations, keywords), and how they are related through a Sankey diagram.

Three-field plot of the keywords plus analysis: (middle field: affiliations; left field: authors; right field: keywords).
